# Advanced myocardial tissue characterisation by a multi-component CMR protocol in patients with rheumatoid arthritis

**DOI:** 10.1007/s00330-017-4838-4

**Published:** 2017-05-12

**Authors:** Simon Greulich, Agnes Mayr, Daniel Kitterer, Joerg Latus, Joerg Henes, Francesco Vecchio, Philipp Kaesemann, Alexandru Patrascu, Andreas Greiser, Stefan Groeninger, Francesco Romeo, Niko Braun, M. Dominik Alscher, Udo Sechtem, Heiko Mahrholdt

**Affiliations:** 1 Division of Cardiology, Robert-Bosch-Medical Center Stuttgart, Stuttgart, Germany; 2 Division of Radiology, University Hospital Innsbruck, Anichstrasse 35, 6020 Innsbruck, Austria; 3 Division of Nephrology, Department of Internal Medicine, Robert-Bosch-Medical Center Stuttgart, Stuttgart, Germany; 4 Centre for Interdisciplinary Clinical Immunology, Rheumatology and Auto-inflammatory Diseases and Department of Internal Medicine II (Oncology, Haematology, Immunology, Rheumatology, Pulmology), University Hospital Tuebingen, Tuebingen, Germany; 5 Division of Cardiology, Universita degli Studi di Roma “Tor Vergata”, Rome, Italy; 6 Siemens Healthcare GmbH, Erlangen, Germany

**Keywords:** Rheumatoid arthritis, Myocardial involvement, Cardiovascular magnetic resonance, Late gadolinium enhancement, Mapping

## Abstract

**Objectives:**

Rheumatoid arthritis (RA) patients are at increased risk of suffering from adverse cardiovascular events. Cardiovascular magnetic resonance (CMR) mapping techniques might be appropriate tools to complement late gadolinium enhancement (LGE) for the assessment of myocardial involvement. This study aimed to perform advanced myocardial tissue characterisation in RA patients by a multicomponent CMR protocol.

**Methods:**

22 RA patients were prospectively enrolled and underwent CMR, including LGE and T1/T2 mapping sequences; 20 volunteers served as controls.

**Results:**

Mean LV-EF was 66%; prevalence of LGE was 18%. RA patients had increased native T1 (985 vs. 959 ms, *p* = 0.03), expanded extracellular volume (ECV) (27 vs. 25%, *p* = 0.02) and higher T2 values (52 vs. 49 ms, *p* < 0.001) compared to controls irrespective of the presence of LGE. T2 mapping showed the highest prevalence of values beyond the 95% percentile of controls.

**Conclusion:**

RA patients demonstrated higher T1, ECV and T2 values compared to controls, with most significant differences for T2. Since these results seem to be independent of the presence of LGE, advanced myocardial tissue characterisation including CMR mapping techniques in addition to LGE-CMR might be useful in the evaluation of myocardial involvement in RA patients.

***Key points*:**

• *RA patients had higher T1*, *ECV and T2 values compared to controls*.

• *Most significant differences were observed for T2*.

• *Our results seem to be independent of the presence of LGE*.

• *Mapping might be useful in the evaluation of myocardial involvement in RA*.

## Introduction

Rheumatoid arthritis (RA) is a systemic inflammatory disorder with articular und extra-articular manifestations, which has a prevalence of 1% in the general population with a preponderance in women [1]. RA may involve the heart, and patients show a reduced average lifespan by 8–15 years primarily due to adverse cardiovascular events [2, 3]. Beside an increased risk of premature atherosclerosis even in the absence of traditional risk factors (RA is regarded as a cardiovascular risk factor equal to diabetes mellitus) [4], ongoing autoimmune, inflammatory and fibrotic processes are supposed to account for (1) symptoms of heart failure, (2) diastolic dysfunction, (3) dilated ventricles with reduced left ventricular ejection fraction (LV-EF) and (4) arrhythmia, resulting in significant cardiovascular co-morbidity and mortality [5–7]. Since patients with RA often run a subclinical course before overt cardiac involvement might manifest [2, 5, 8, 9], non-invasive advanced tissue characterisation in early, potential modifiable stages of the disease is highly desirable. T1 and T2 CMR mapping sequences, which perform well in the detection of subtle myocardial fibrotic/inflammatory processes [10–12], and assess quantitatively myocardial tissue properties in absolute terms, might complement the performance from established late gadolinium enhancement (LGE)-CMR, for the evaluation of cardiac involvement in RA patients.

Hence, aim of our study was to perform advanced myocardial tissue characterisation by a multicomponent CMR protocol, including LGE and T1/T2 mapping sequences, for evaluation of myocardial involvement in patients presenting with RA.

## Methods

### Patient population

Twenty-two RA patients without history of coronary artery disease (CAD) presenting at our institution between October 2013 and May 2016 were consecutively enrolled and underwent a multicomponent CMR protocol. Exclusion criteria were contraindications for CMR (e.g. pregnancy, pacemaker/ICD, glomerular filtration rate less than 30 mL/min, previous adverse reactions to gadolinium, cochlea implant).

Healthy volunteers (*n* = 20) with no history of cardiac disease and free of symptoms served as the control group. Prior to CMR, all participants provided a blood sample for measurement of haematocrit. The ethics committee of the University of Tuebingen approved the study, and all patients gave written informed consent.

### CMR protocol

ECG-gated CMR was performed in breath-hold using a 1.5-T MAGNETOM Aera (Siemens Healthcare, Erlangen, Germany) in line with current recommendations [13]. Both cine and LGE short-axis images were prescribed every 10 mm (slice thickness 6 mm) from base to apex. In-plane resolution was typically 1.2 × 1.8 mm. Cine was performed using a steady-state free-precession (SSFP) sequence. LGE images were acquired on average 5–10 min after contrast using a segmented inversion recovery gradient echo (IR-GRE) sequence constantly adjusting inversion time to null normal myocardium [14]. The contrast dose (gadopentetate dimeglumine) was 0.15 mmol/kg.

A modified look-locker inversion recovery prototype sequence (MOLLI) was used for T1 mapping and performed in a single midventricular short-axis (SAX) slice at mid-diastole, prior to and 20 min after administration of contrast, in line with current recommendations [15].

Short-axis T2 mapping was performed in a matching midventricular SAX before administration of contrast agent using an ECG-triggered T2-prepared single-shot balanced SSFP prototype sequence with multiple T2 preparation times [16].

Detailed information on T1 and T2 mapping sequences is provided in the Appendix.

### CMR analysis

Cine and LGE images were evaluated by experienced observers (S.G., H.M.) as described elsewhere [17]. In brief, endocardial and epicardial borders were outlined on the short-axis cine images. Volumes, mass and ejection fraction were derived by summation of epicardial and endocardial contours. The distribution of LGE was characterised as epicardial, intramural, transmural or subendocardial [17].

Colour-coded T1, ECV and T2 maps were generated on the basis of inline-generated, motion-corrected raw images using QMap software 1.0 (Medis, Leiden, the Netherlands) in a single matching midventricular SAX. Motion-corrected T1 maps were examined for quality in three modalities: (1) raw T1 images, (2) T1 maps, (3) *R* ^2^ maps. Endo- and epicardial contours were manually drawn by two experienced observers (S.G., A.M.), and then divided into six segments using the anterior right ventricular insertion point as reference. Care was taken to avoid partial volume effects at the endocardial and epicardial borders for T1, ECV and T2 maps. Global T1, ECV and T2 values were calculated: T1 values were determined by fitting an exponential model to the measured data [18]. Prior to CMR, the haematocrit was determined in all subjects, allowing with native and post contrast T1 measurements of the myocardium and blood pool the calculation of extracellular volume (ECV), using a previously described equation [19]. T2 results were obtained by fitting a two-parameter intensity-weighted exponential model (no offset term) [20].

### Variables and definitions

All variables were collected directly from patients and/or medical records except CMR parameters, which were evaluated as described above.

Patients with RA had to fulfil the diagnostic criteria of the American College of Rheumatology [21].

Evaluation of disease activity in RA patients used the Disease Activity Score 28 (DAS-28) [22].

### Statistical analysis

Absolute numbers and percentages were computed to describe the patient population. All continuous variables were tested for normality using the Kolmogorov–Smirnov test. Normally distributed continuous variables were expressed as means (with standard deviation) and skewed variables were presented as medians (with quartiles). Comparisons between groups were made using the Mann–Whitney *U* test or the Fisher’s exact test, as appropriate. *P* values (two-tailed) of less than 0.05 were considered significant. Bivariate correlations were assessed by using the Pearson’s or the Spearman’s coefficient, as appropriate. Intra- and interobserver variability was assessed via intraclass correlation coefficients (ICC). All statistical analyses were performed using SPSS, version 22.0 (IBM Corp., Armonk, NY, USA).

## Results

### Baseline characteristics

Overall, 42 subjects were studied: 22 RA patients and 20 healthy controls who did not differ significantly regarding age and gender (*p* = 0.09 and *p* = 0.66, respectively). RA patients were 63 ± 12 years of age, with a female preponderance (64%).

Nonspecific dyspnoea and angina were the most frequently reported symptoms in the RA patient population (41% and 27%, respectively). ECG abnormalities occurred in 32% of the RA patients (*n* = 7): *n* = 3 atrial fibrillation, *n* = 2 ventricular extrasystoles, *n* = 1 supraventricular extrasystoles, *n* = 1 T wave abnormality. Mean DAS-28 was 3.4, reflecting active disease in the majority of patients. Consequently, most patients were under immunosuppressive medication during time of CMR (73% on steroids, 27% on methotrexate). More details can be viewed in Table 1.

**Table 1:** Baseline patient characteristics

Age (years)	63 ± 12
Gender (male)	8 (36%)
Cardiovascular risk factors	
Diabetes	2 (9%)
Hypertension	13 (59%)
Smoking^a^	12 (55%)
Hyperlipidaemia	8 (36%)
Family history of CVD	7 (32%)
Obesity (BMI ≥ 30 kg/m^2^)	7 (32%)
Symptoms (multiple possible)	
Angina	6 (27%)
Dyspnoea	9 (41%)
Palpitations	2 (9%)
Syncope	–
ECG abnormality	7 (32%)
Years since diagnosis	
	3 (2–10)
< 1	3 (14%)
1–4	10 (45%)
5–9	3 (14%)
> 10	6 (27%)
Disease activity	
DAS-28	3.4 (1.8–6.2)
Haematocrit	0.38 (0.34–0.42)
Medication	
Beta-blockers	7 (32%)
ARB	12 (54%)
ASA	2 (9%)
CCB	8 (36%)
Statins	5 (23%)
Diuretics	4 (18%)
Steroids	16 (73%)
NSAID	4 (18%)
Antibodies	2 (9%)
Cyclophosphamide	2 (9%)
Azathioprine	1 (4%)
Methotrexate	6 (27%)

All values are *n* or mean SD or median interquartile ranges

*CVD* cardiovascular disease, *BMI* body mass index, *ECG* electrocardiogram, *ARB* angiotensin receptor blockers, *ASA* acetylsalicylic acid, *CCB* calcium channel blockers, *NSAID* nonsteroidal anti-inflammatory drug

^a^Current or ever smokers

### Functional and LGE CMR results

CMR findings are displayed in Table 2. We observed no significant difference in left ventricular (LV) size or ejection fraction (EF) between RA patients and controls. Of note, LV-EF was preserved (67 ± 6 in RA patients vs. 66 ± 10 in controls, *p* = 0.76). Left atrial (LA) size was larger in RA patients than in controls (*p* = 0.05). LGE occurred rarely: four of the RA patients (18%) and none of the controls showed LGE. Ischaemic-type LGE pattern could be detected in none of the RA patients; all LGE patterns were classified as non-ischaemic-type origin (epicardial or intramural) [17].

**Table 2:** CMR findings

	Controls (*n* = 20)	Patients (*n* = 22)	*p*
LV-EF (%)	67 ± 6	66 ± 10	0.76
LV-EDV (ml)	103 ± 29	112 ± 37	0.65
LV-ESV (ml)	35 ± 14	41 ± 32	0.91
LV-SV	69 ± 16	71 ± 13	0.43
LV-EDD	44 ± 6	46 ± 6	0.48
LA (cm^2^)	20 ± 3	22 ± 4	**0.05**
IVS (mm)	10 ± 2	11 ± 2	0.07
PA (mm)	24 ± 4	24 ± 3	0.77
LGE per patient	-	4 (18%)	
Extent (g)/% LV mass	–	2.3 (2%)	
Epicardial	–	1 (4%)	
Intramural	–	3 (14%)	
Transmural	–	–	
Subendocardial	–	–	
Native T1 (ms)	959 (923–978)	985 (946–1009)	**0.03**
Post contrast T1 (ms)	524 (509–550)	508 (485–536)	0.14
ECV (%)	25 (23–26)	27 (25–31)	**0.02**
T2 (ms)	49 (48–51)	52 (50–54)	<**0.001**

All values are mean ± SD or median interquartile ranges

Bold values indicate significant *p*-values. *P*-values (two-tailed) of <0.05 were considered significant

*CMR* cardiac magnetic resonance, *LV* left-ventricular, *EF* ejection fraction, *EDV* end-diastolic volume, *ESV* end-systolic volume, *SV* stroke volume, *EDD* end-diastolic diameter, *LA* left atrium, *IVS* interventricular septum, *PA* pulmonary artery, *LGE* late gadolinium enhancement, *ECV* extracellular volume

### T1 and ECV results

**Fig. 1:**
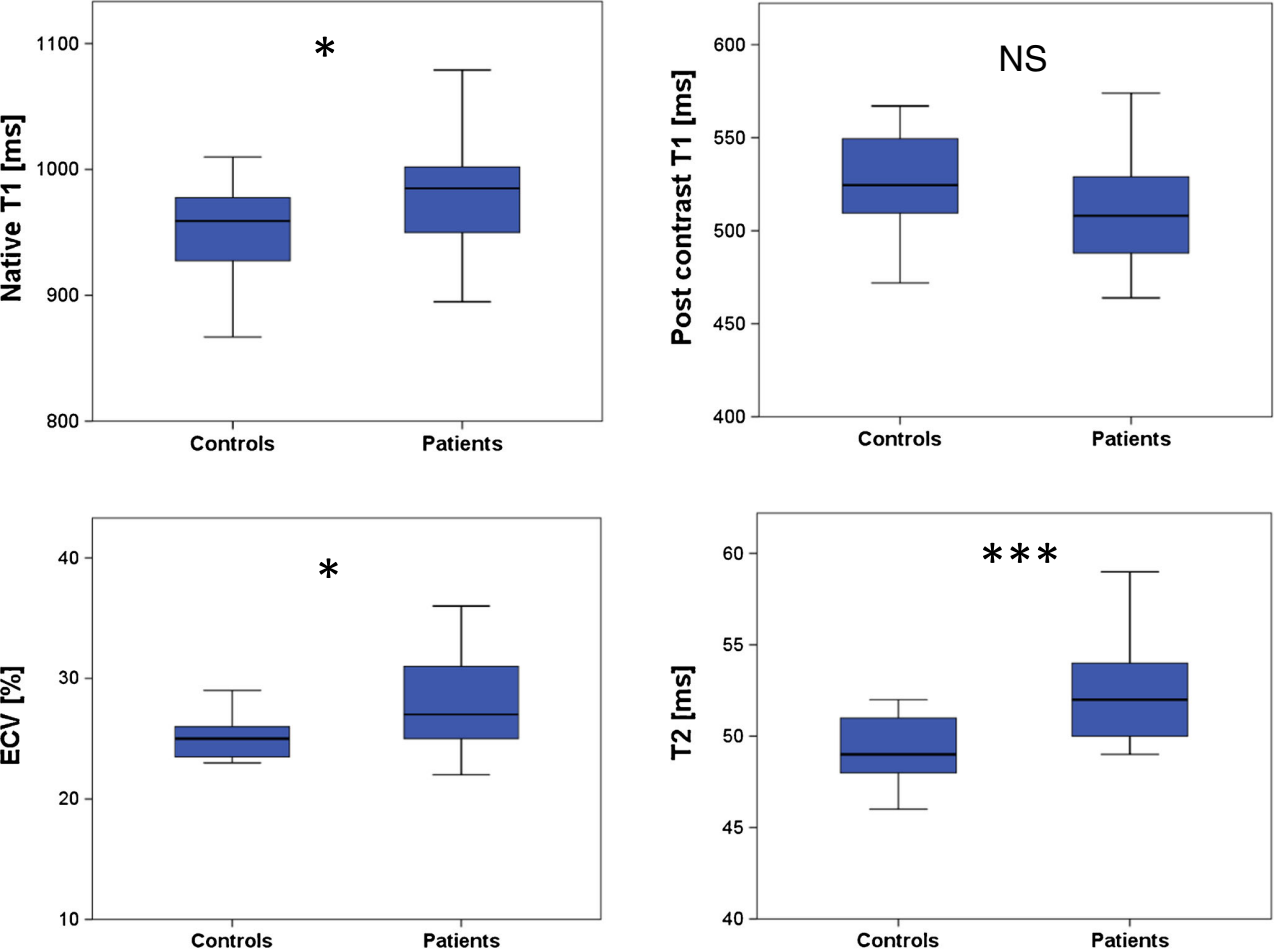
Box plots for median native T1, post contrast T1, extracellular volume fraction (ECV), and T2 mapping in controls and RA patients; the *centre line* in each box represents the median, whereas the *lower* and *upper limits* of each box represent the 25th and 75th percentiles, respectively. Except for post contrast T1, RA patients showed values which were significantly different to the values of the control group: *p ≤ 0.05; ***p ≤ 0.001

RA patients showed significantly higher native T1 values 985 (946–1009) ms than controls 959 (923–978) ms (*p* < 0.03); also see Table 2 and Fig. 1. In contrast, post contrast T1 values did not differ significantly to those in controls: 508 (485–536) ms vs. 524 (509–550) ms (*p* = 0.14). Expanded ECV was observed in RA patients: 27 (25–31)% vs. 25 (23–26)% in the control group (*p* < 0.02). Dividing our RA patients into two groups according to the presence of LGE (LGE positive vs. LGE negative) revealed that native T1 values, post contrast T1 values and ECV values were not significantly different between both groups (*p* = 0.28, *p* = 0.13, *p* = 0.18, respectively).

We demonstrated excellent intra- and interobserver agreement in T1 and ECV values (ICC for intraobserver agreement was 0.98 each for native T1, post contrast T1 and ECV. ICC for interobserver agreement was 0.98 for native T1, 0.97 for post contrast T1 and 0.98 for ECV).

In our RA patients with ECG abnormalities (*n* = 7), native and post contrast T1 values as well as ECV did not differ to the values in RA patients with unremarkable ECG (*p* = 0.44, *p* = 0.48, *p* = 0.85, respectively).

Looking at patients’ disease activity (DAS-28) revealed no significant correlation to the values of native T1, post-contrast T1 and ECV. Likewise, there was no significant correlation in RA patients between duration of disease and native, post contrast T1 and ECV values (all *p* > 0.05).

## T2 results

Median myocardial T2 values were significantly higher in patients than in controls: 52 (50–54) ms vs. 49 (48–51) ms (*p* < 0.001; Table 2 and Fig. 1). Again, values between LGE-positive and LGE-negative patients did not differ significantly (*p* = 0.80). Similar to T1 and ECV values, we found excellent intra- and interobserver agreement for T2 values (ICC 0.99 for intraobserver agreement and 0.98 for interobserver agreement). T2 values in patients with ECG abnormalities were not significantly different from T2 values in patients with normal ECG (*p* = 0.67).

Furthermore, T2 values of RA patients did not correlate with disease activity (DAS-28) (*p* = 0.08). However, T2 values were associated with disease duration (*R* = 0.51, *p* = 0.02).

**Fig. 2:**
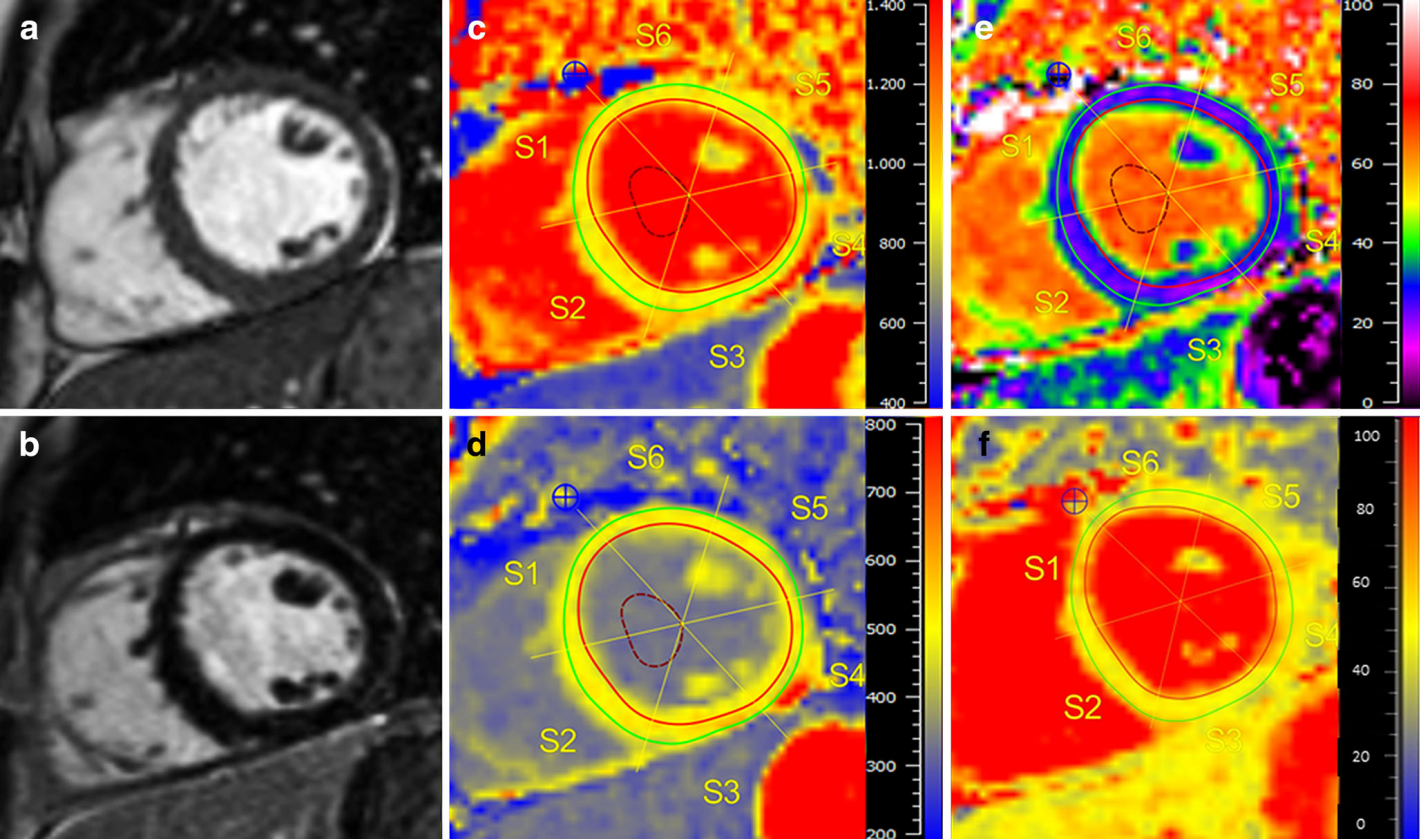
RA patient with no LGE but abnormal mapping values. Cardiac magnetic resonance (CMR) of a 50-year-old woman suffering from RA with no history of dyspnoea or angina and normal ECG: Cine images (**a**) showed a normal LV-EF (66%), LGE images (**b**) revealed no enhancement. Native T1 map (**c**) showed an increased T1 with 1004 ms (normal median range 959 (923–978) ms), decreased post-contrast T1 (**d**) with 513 ms (normal median range 524 (509–550) ms) and expanded ECV (**e**) of 28% (normal median range 25 (23–26)%). T2 (**f**) was prolonged at 54 ms (normal median range 49 (48–51) ms)

Our findings are illustrated in Fig. 2, displaying an LGE-negative RA patient with increased values for native T1, ECV and T2 compared to values from healthy controls.

### Values above the 95% percentile of normal

**Fig. 3:**
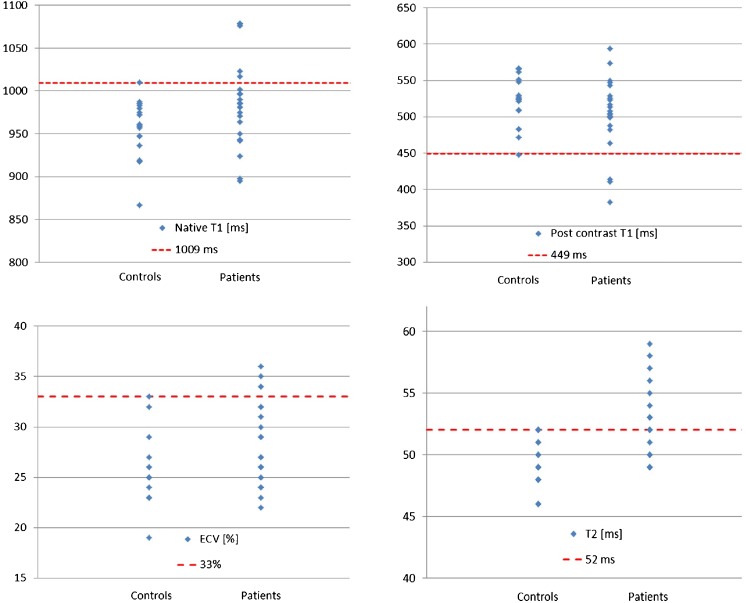
Values above the 95% percentile of normal. Values for T1 (native, post contrast), ECV and T2 in controls and RA patients with *dotted lines* indicating values beyond the 95% percentile of the control group considered as definite abnormal values (1009 ms for native T1, 449 ms for post contrast T1, 33% for ECV and 52 ms for T2). *Some of the values might be similar, with dots overlapping one another

Since RA patients and controls showed significant overlap in mapping values (also see Fig. 1), we defined the 95% percentile of our control group as a threshold for definite abnormal values. Thus, values above 1009 ms for native T1, below 449 ms for post contrast T1, above 33% for ECV and above 52 ms for T2 were found to be definite abnormal (beyond the 95% percentile of normal controls) (see Fig. 3). We observed no significant difference in age between RA patients beyond and within the 95% percentile regarding our mapping results.

In detail, 23% (*n* = 5/22) of the RA patients demonstrated a native T1 value above the 95% percentile of our matched control group (Fig. 3). Four patients (18%) showed post contrast values below the 95% percentile, and 4 (18%) patients had expanded ECV above the 95% percentile of controls. The majority of definite abnormal values were observed for T2 values, with 41% (9/22) of the RA patients, underscoring a substantial grade of inflammation in the RA patient population. Out of these nine patients with definite abnormal T2 values, four patients also showed definite abnormal values for native T1, and two showed definite abnormal values for both post contrast T1 and ECV, respectively.

Most patients with values beyond the 95% percentile of controls were LGE negative: (a) 80% (4/5 patients) for native T1, (b) 50% (2/4 patients) for post contrast T1, (c) 75% (3/4 patients) for ECV, (d) 78% (7/9 patients) for T2.

## Discussion

This study is unique in performing advanced myocardial tissue characterisation by a multicomponent CMR protocol including LGE-CMR, and T1/T2 mapping in RA patients. First, we found that, despite a low prevalence of LGE, a significant proportion of RA patients show diffuse myocardial inflammation and fibrosis by mapping techniques on CMR. Second, T2 was the parameter which best separated RA patients and controls, and of all mapping parameters it showed the highest percentage of values above the 95% percentile of controls.

### Baseline characteristics and general CMR results

The majority of RA patients were female and asymptomatic to mildly symptomatic [5] (Table 1). Although dyspnoea was reported in 41% of the RA patients, it is difficult to separate dyspnoea suggestive of cardiac origin from dyspnoea due to low physical activity, since RA patients are often severely affected by articular and extra-articular manifestations, hampering them in their daily lives [5]. Similar to previous studies [23, 24], mean LV-EF was preserved (66%), and LA size enlarged compared to controls, suggestive of diastolic dysfunction, which is a common finding in RA patients [24] (Table 2). Overall, 18% of the RA patients demonstrated non-ischaemic-type LGE, which is lower than in other reports [23, 24]. The difference might be explained by different disease duration between the studies. In this study, median disease duration was 3 years whereas in the study by Ntusi et al. [24] it was 7 years. Accordingly, Kobayashi et al. [23] found that in LGE-positive RA patients median RA duration was greater, suggesting that our patients were in a relatively early stage of the disease, in which myocardial alterations might still be potentially reversible before irreversible scar (as detected by LGE) might occur.

### T1 and ECV results

RA patients showed significantly higher native T1 values and ECV values than controls (*p* = 0.03 and *p* = 0.02, respectively; Table 2, Fig. 1). These increased values for native T1 and ECV represent most likely, in absence of inflammation, amyloidosis or other forms of infiltrative myocardial disease, diffuse myocardial fibrosis [15], which cannot be detected by LGE-CMR, but is a well-known histologic finding in patients with RA, potentially leading to significant cardiovascular co-morbidity [5]. Interestingly, native T1 values, post contrast T1 values and ECV values were not significantly different between LGE-negative and LGE-positive groups (*p* = 0.28, *p* = 0.13, *p* = 0.18, respectively), underscoring additional diagnostic value to the performance of LGE-CMR alone in patients with RA. These results are in line with the results from Ntusi et al. [24]. They investigated 39 RA patients by CMR including native T1, ECV and LGE sequences. However, in contrast to the latter study, in which post contrast T1 values showed a weak but significant difference to controls (*p* = 0.04), the post contrast T1 values in our study showed no significant difference between RA patients and controls (*p* = 0.14), which might be due to the lower patient number in our study. Anyway, since post contrast T1 values depend on several factors, including native T1, type and dosage of gadolinium contrast applied, and the post contrast acquisition time within the contrast pharmacodynamics redistribution process [25], native T1 and ECV are the currently preferred parameters for myocardial T1 quantification [15].

Almost a third of the RA patients (32%, *n* = 7) demonstrated ECG abnormalities. Nevertheless, native and post contrast T1 values and ECV did not differ to the values in RA patients with unremarkable ECG, reflecting the difficulties in the cardiac evaluation of RA patients by a “standard” cardiac assessment, and the potential additive value of advanced myocardial tissue characterisation by a multicomponent CMR protocol, which might be repeated arbitrarily not only for diagnosis but also for monitoring of cardiac involvement in RA.

## T2 results

T2 is prolonged in edematous, inflammatory myocardial tissue [26]. Compared to other studies, which used T2-weighted images with all their known limitation for assessment of myocardial inflammation [24], this study, as a new finding, performed T2 mapping as a quantitative component of a multiparametric CMR protocol. T2 mapping is a recent and robust technique that performs well in inflammatory states such as myocarditis, showing good correlation to histologic findings [11, 27]. Median myocardial T2 values were significantly higher in RA patients than in controls, and T2 seems to represent the best CMR parameter in our protocol to separate RA patients from controls (*p* < 0.001), suggesting a significant proportion of myocardial inflammation in our RA patients (Table 2, Fig. 1). Likewise to T1 mapping parameters, T2 values (1) did not differ significantly between LGE-positive and LGE-negative patients (*p* = 0.80) and (2) were not significantly different in patients with ECG abnormalities from patients with normal ECG (*p* = 0.67).

Ntusi et al. [24] performed T2-weighted images instead of recent T2 mapping in their study with RA patients. Hence, their finding of a non-significant difference in the overall global myocardial T2 signal intensity ratio between RA subjects and controls might be a limitation of the used sequence rather than a reliable description of the patients’ myocardial inflammatory status. Another potential explanation for these discrepant findings might be the fact that our group had shorter time duration since onset of RA (3 years), with a rather high rate of steroids but lower rate of methotrexate medication, and therefore potentially less controlled inflammation yielding to increased T2 values.

T2 values were associated with RA disease duration (*R* = 0.51, *p* = 0.02), suggesting a higher grade of inflammation with ongoing disease activity in RA patients. It is important to keep in mind that these differences of T2 in absolute terms are only detectable by recent T2 mapping techniques. If further studies could reveal that mapping values are influenced by the patients’ immunosuppressive medication, which seems obvious, T2 (and T1) mapping could represent a reliable biomarker for monitoring of the clinical course in patients with RA.

Our findings are illustrated in Fig. 2, which shows a LGE-negative RA patient with increased values for native T1, ECV and T2.

### Values above the 95% percentile of normal

Since RA patients and controls showed overlap in mapping values (Fig. 1), we defined the 95% percentile of our control group as a threshold for definite abnormal values (Fig. 3). Similar to the non-significant difference regarding age between control group and RA patient group, we observed no significant difference in age between RA patients with mapping values beyond and within the 95% percentile. Therefore, we think that the highly significant differences in mapping values reported in our study are not primarily driven by age.

The majority of definite abnormal values were observed for T2 values (41%, 9/22 of the RA patients), underscoring a substantial grade of inflammation in the RA patient population despite immunosuppressive therapy (Fig. 3). Out of these nine patients with definite abnormal T2 values, four patients also showed definite abnormal values for native T1, and two showed definite abnormal values for both post contrast T1 and ECV, illustrating the coincidence of diffuse myocardial inflammation and diffuse myocardial fibrosis. With the majority of RA patients classified LGE-negative, these myocardial alterations would have been undetected otherwise, highlighting the value of an advanced myocardial tissue characterisation by a multicomponent CMR protocol, including LGE and T1/T2 mapping sequences, in patients with RA.

### Clinical implications

This study could show that advanced myocardial tissue characterisation by a multicomponent CMR protocol, including established LGE and recent T1 and T2 mapping sequences, might be reasonable in patients with RA. We found increased values for native T1, ECV and T2 in RA patients compared to controls, with the most significant difference for T2 mapping values, underscoring a substantial part of myocardial inflammation and fibrosis in these patients. Since clinical presentation and standard cardiac work-up including ECG and echo are often inconclusive, advanced tissue characterisation by (a) measuring parameters in absolute terms and (b) potential for serial follow-up due to the lack of radiation might be an appropriate approach in these patients, who run a high risk of suffering from cardiovascular morbidity and mortality.

Despite these encouraging results, multicentre randomised trials are needed to define further the diagnostic and prognostic role of abnormal mapping findings in RA patients, before these sequences should be used in clinical routine.

### Limitations

Some limitations in this study have to be addressed. Since this is a single-centre setting, potential centre-specific bias cannot be excluded. On the other hand, mapping sequences are vendor- and site-specific, so each site should create its own reference values based upon healthy controls, as suggested by current recommendations [15].

The number of RA patients is small. However, many studies dealing with RA show a comparable number of patients, and as a result of significant differences in comparison to controls, the patient number seems appropriate.

Although measurement of myocardial T1 or T2 values in the entire midventricular slice might tend to miss local abnormalities, this approach is widely used [28–30], more objective than a regional approach and swiftly comparable to follow-up examinations.

Endomyocardial biopsy (EMB) was not performed. However, EMB suffers from several limitations, e.g. invasiveness, sampling error, thereby lowering its diagnostic benefit. Furthermore, in RA subjects with nonspecific symptoms and normal LV-EF, this approach would not correspond to current guidelines [17].

Comparing mapping results to cardiac biomarkers would have been of interest; however, this was not intention of our study, and should be investigated by further studies.

Finally, we have no follow-up data in these patients and we thus do not know about the additive predictive value of the mapping sequences employed in the study.

## Conclusions

Our study revealed increased values for native T1, ECV and T2 in RA patients compared to controls, with the most significant difference for T2 mapping values. Furthermore, these results were independent of the presence of LGE and suggest a substantial component of diffuse myocardial inflammation and fibrosis in RA patients. Since clinical presentation and standard cardiac work-up are often inconclusive and face high cardiovascular morbidity and mortality in RA patients, advanced tissue characterisation with a combination of LGE and recent mapping techniques might be an adequate approach for both detection and monitoring of myocardial involvement.

Nevertheless, multicentre randomised trials are needed to evaluate further the diagnostic and prognostic value of T1 and T2 mapping in RA patients, before these sequences can enter the clinical setting.

## Appendix

### CMR protocol

#### T1 Mapping

T1 mapping was performed in short axis orientation using a modified Look-Locker inversion recovery (MOLLI) sequence before and 20 minutes after contrast media administration.

Three inversion episodes were employed, where in the first episode 3 images were acquired, followed by 3 recovery heartbeats, then 3 images in the second episode and another image in the 3^rd^ episode (3(3)3(0)1 MOLLI acquisition scheme), resulting in 7 images in total and a scan duration of 10 heartbeats.

Inversion times (TI) were 120, 200, 280 ms respectively in the initial images after inversion,

Typical imaging parameters were TE/TR 1.0/2.4 ms, acquisition time per heartbeat 168 ms, flip angle 35°, bandwidth 1371 Hz/pixel, matrix 192 × 136 pixels, measured in-plane spatial resolution 1.9 × 2.0 mm² and slice thickness 8 mm, PAT acceleration R = 2.

#### T2 Mapping

T2 mapping was performed in short axis orientation before administration of contrast media using a T2-prepared single-shot bSSFP prototype sequence. T2-weighted images were obtained using ECG gating to diastole in a single breath-hold with 0, 25, and 55 ms T2 preparation times. Motion correction based on elastic registration was performed allowing generation of T2 pixel maps. After each single image acquisition, 4 heartbeats were used for signal recovery.

Typical imaging parameters were TE/TR 1.1/2.5 ms, acquisition time per heartbeat 150 ms, flip angle 70°, bandwidth 1445 Hz/pixel, matrix 192 × 116 pixels, measured in-plane spatial resolution 1.9 × 2.3 mm² and slice thickness 8 mm, PAT acceleration R = 2.

## Abbreviations

CMR: cardiovascular magnetic resonance
CVD: cardiovascular disease
ECG: electrocardiogram
ECV: extracellular volume
EMB: endomyocardial biopsy
IQR: interquartile range
IVS: interventricular septum
LA: left atrium
LGE: late gadolinium enhancement
LV: left ventricle
LV-EDV: left ventricular end-diastolic volume
LV-EF: left ventricular ejection fraction
LV-ESV: left ventricular end-systolic volume
MOLLI: modified look-locker inversion recovery sequence
RA: rheumatoid arthritis
SCD: sudden cardiac death
SSFP: steady-state free-precession
